# Hyaluronic Acid-Based Biomaterials in Tissue Engineering: From Molecular Properties to Re-Generative Applications

**DOI:** 10.3390/jfb17050246

**Published:** 2026-05-14

**Authors:** Chao-Ming Su, Ming-You Shie, Wan-Ni Huang, Fang-Jou Chiu, Hong-Kai Chen, Yi-Wen Chen, Yu-Fang Shen

**Affiliations:** 1 Advanced Therapeutic & Pharmaceutical Center, China Medical University Hospital, Taichung City 40447, Taiwan; suchaoming@gmail.com (C.-M.S.); w123wanni@yahoo.com.tw (W.-N.H.); hankel.tw@gmail.com (H.-K.C.); 2 Xenotransplantation Translational Research Center, China Medical University Hospital, Taichung City 40447, Taiwan; eviltacasi@gmail.com; 3 School of Dentistry, China Medical University, Taichung City 40447, Taiwan; 4 Department of Bioinformatics and Medical Engineering, Asia University, Taichung City 41354, Taiwan; 98130crystal@gmail.com; 5 Graduate Institute of Biomedical Sciences, China Medical University, Taichung City 40447, Taiwan; 6 High Performance Materials Institute for xD Printing, Asia University, Taichung City 41354, Taiwan

**Keywords:** hyaluronic acid, hydrogel modification, tissue engineering, regenerative medicine, HA binding receptor, 3D bioprinting

## Abstract

Hyaluronic acid (HA), a native non-sulfated glycosaminoglycan of the extracellular matrix, has emerged as a central biomaterial in tissue engineering due to its biocompatibility, hydration capacity, and receptor-mediated bioactivity. Beyond its structural role, HA actively regulates cellular behaviors through interactions with receptors such as CD44 and RHAMM, with outcomes highly dependent on molecular weight, degradation state, and matrix context. Recent advances in chemical modification and crosslinking strategies have enabled the development of HA-based hydrogels, nanofibers, and composite systems with tunable mechanics and degradation profiles, supporting applications in bone, cartilage, vascular, and skin regeneration, as well as in emerging platforms such as 3D bioprinting and nanomedicine. However, inconsistent biological responses and limited clinical translation remain key challenges. This review integrates current understanding of HA synthesis, physicochemical properties, degradation, and receptor-mediated signaling, and establishes a mechanistic framework linking molecular characteristics, matrix mechanics, and cell responses. Building on this framework, we outline design strategies for multifunctional HA composites, advanced biofabrication approaches, and receptor-targeted systems, providing a basis for the rational engineering of next-generation HA-based biomaterials with improved translational potential.

## 1. Introduction

Tissue injury and organ damage remain major challenges in modern healthcare. The growing demand for organ transplantation, the limited availability of suitable donors, and the requirement for long-term immunosuppressive therapy have driven the development of alternative therapeutic strategies [1]. In this context, tissue engineering and regenerative medicine have emerged as promising approaches for repairing or replacing damaged tissues and organs. Over the past three decades, these interdisciplinary fields—integrating biology, engineering, and materials science—have focused on the development of biomaterials and bioactive constructs that can restore tissue structure and function [2].

In tissue engineering, functional tissues can be regenerated by delivering cells, growth factors, or bioactive scaffolds to damaged sites. Among these components, scaffolds play a crucial role because they act as temporary artificial extracellular matrices (ECMs), providing structural support and guiding cell adhesion, proliferation, migration, and differentiation during tissue regeneration [3,4].

Polymeric materials are widely considered ideal scaffold candidates for tissue engineering applications because their physicochemical properties can be tailored to meet specific biological requirements, including biocompatibility, mechanical strength, and controlled biodegradability. These materials are generally classified as synthetic or natural polymers [5]. Synthetic polymers, such as poly(N-isopropylacrylamide) (PNIPAM) [6] and poly(ethylene glycol) (PEG) [7], have been extensively investigated due to their tunable mechanical and physicochemical properties. In contrast, natural polymers are often preferred in regenerative medicine because they more closely resemble native extracellular matrices and exhibit excellent biocompatibility and biodegradability. Common natural biomaterials used in hydrogel fabrication include collagen, alginate, chitosan, fibrin, chondroitin sulfate, and hyaluronic acid (HA) [8].

Among natural polysaccharides, HA is particularly distinctive because it is a native component of the mammalian extracellular matrix rather than merely an exogenous biomaterial [8,9]. Structurally, HA is the only non-sulfated glycosaminoglycan and exists as a free linear polymer instead of being covalently attached to a core protein [10,11]. Functionally, HA exhibits exceptional water-binding capacity, viscoelasticity, and space-filling properties, enabling it to generate highly hydrated microenvironments that support lubrication, nutrient transport, and mechanical buffering [11,12]. More importantly, unlike many polysaccharides that mainly act as passive structural materials, HA can actively regulate cell migration, proliferation, inflammation, and tissue remodeling [13] through specific receptors such as CD44 and RHAMM [14,15]. In addition, its biological effects are strongly dependent on molecular weight, with high-molecular-weight HA generally associated with tissue protection and low-molecular-weight HA fragments often linked to inflammatory signaling [16]. These unique structural and biological properties make HA particularly attractive for tissue engineering and regenerative medicine [17,18].

However, despite extensive progress, a systematic understanding of how HA molecular characteristics, matrix properties, and receptor-mediated signaling collectively govern cellular responses remains lacking. This review aims to integrate these aspects and provide a mechanistic framework to guide the rational design of HA-based biomaterials.

## 2. HA Synthesis, Properties and Degradation

### 2.1. Synthesis

HA is synthesized by three kinds of transmembrane enzymes, namely HA synthase 1 (HAS1), HA synthase 2 (HAS2), and HA synthase 3 (HAS3), on the inner side of the plasma membrane [19]. HA is synthesized by hyaluronan synthases through the alternating addition of UDP-glucuronic acid and UDP-N-acetylglucosamine. The elongating HA polymer is concurrently translocated across the plasma membrane into the extracellular matrix, potentially involving ATP-binding cassette (ABC) transporter systems [20]. The three HAS isoforms (HAS1, HAS2, and HAS3) exhibit approximately 50–70% sequence similarity, yet they are encoded by different genes located on separate chromosomes [21]. Under different physiological and pathological conditions, the expression and activity of HAS isoforms vary, resulting in the production of HA with different molecular weights. HAS1 and HAS2 have moderate activity and are responsible for synthesizing high molecular weight HA; HAS3 has the highest activity, responsible for polymerizing low molecular weight HA [22]. In addition, HA exhibits rapid metabolic turnover in vivo. In human skin, the half-life of HA in the dermis is approximately one day, whereas its degradation rate varies considerably among different tissues [23].

### 2.2. Properties

**Figure 1:**
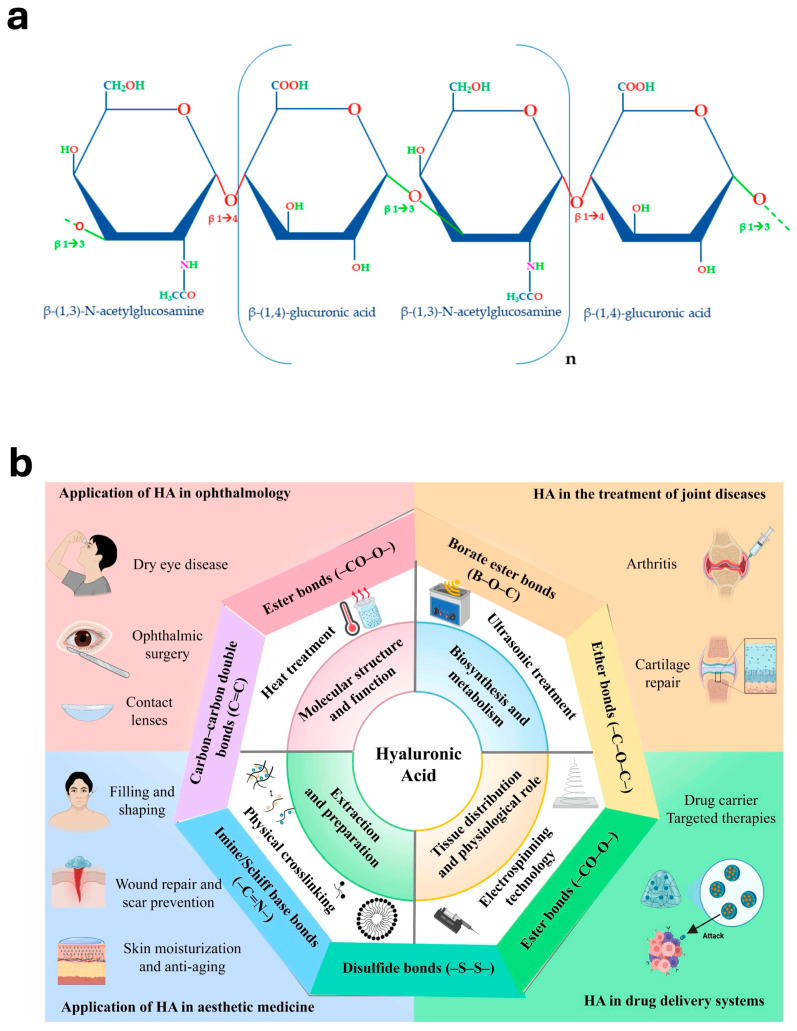
Structural features and functional versatility of HA. (**a**) HA is a linear polysaccharide composed of repeating disaccharide units of N-acetylglucosamine and glucuronic acid linked by alternating β-(1→3) and β-(1→4) glycosidic bonds [9]. Licensed under CC-BY-NC 4.0. (**b**) Representative HA modification chemistries and their relevance to diverse biomedical applications, including tissue repair, ophthalmology, joint regeneration, aesthetic medicine, and drug delivery [25]. Licensed under CC-BY-NC 4.0.

HA is a linear polysaccharide composed of repeating disaccharide units of D-glucuronic acid (GlcA) and N-acetyl-D-glucosamine (GlcNAc), linked by alternating β-1,3 and β-1,4 glycosidic bonds. HA polymers typically contain approximately 2000–25,000 repeating units and can reach molecular weights of 10^3^–10^4^ kDa, with fully extended chains reaching lengths of up to 25 μm (Figure 1a) [9]. HA is widely found in the human body due to its hydrophilic properties. In the extracellular matrix, HA acts as a space-filling macromolecule that creates hydrated environments for cell migration. By increasing interstitial space, HA can reduce cell–cell interactions, modulate cellular communication, and promote tissue remodeling [24]. HA possesses unique viscoelastic properties that arise from its polyelectrolyte nature. The rheological behavior of HA is influenced by electrostatic repulsion between carboxylate groups along the polymer backbone, double-helix conformations of the chains, and intra- and intermolecular hydrogen bonding interactions [12].

HA is highly hydrophilic because its repeating disaccharide units contain abundant hydroxyl, carboxyl, and acetamido groups that strongly interact with water molecules. This extensive hydration enables HA to retain large amounts of water and form highly swollen matrices, which is essential for lubrication and nutrient diffusion in native tissues [26]. In addition, the viscoelastic behavior of HA depends strongly on its molecular weight, concentration, and surrounding ionic environment. Higher-molecular-weight HA generally exhibits greater chain entanglement and stronger intermolecular interactions, resulting in increased solution viscosity and elastic behavior. By contrast, lower-molecular-weight HA shows reduced viscosity and weaker mechanical buffering capacity [12]. Ionic strength can also modulate HA chain conformation by screening electrostatic repulsion between negatively charged carboxylate groups, thereby affecting chain expansion, rheology, and gel behavior [27]. These physicochemical features are particularly important in tissue engineering because they influence scaffold hydration, injectability, mechanical performance, and the ability to mimic native extracellular matrix microenvironments.

In addition to the physical properties of HA, its chemical properties also make it a versatile biomaterial for biomedical and tissue engineering applications. After chemical modification of hydroxyl, carboxyl, and N-acetyl amino terminals, HA is absorbed slowly by the human body, making it suitable for application in tissue engineering [25,28]. Figure 1b illustrates the functional versatility of HA by summarizing its representative chemical bonding modes and their corresponding biomedical applications. Owing to the presence of multiple reactive functional groups, HA can be modified through ester bonds, ether bonds, borate ester bonds, carbon–carbon double bonds, disulfide bonds, and imine/Schiff-base linkages, which expand its structural diversity and enable broad biomedical use. These modification strategies support the development of HA-based materials for ophthalmic applications, treatment of joint diseases, aesthetic medicine, wound healing, and drug delivery systems. Therefore, this figure highlights that the unique molecular structure of HA not only determines its intrinsic physicochemical properties, but also provides a versatile platform for chemical functionalization and application-specific biomaterial design. Esterification and cross-linking are two chemical modifications of HA. Esterification is commonly used to introduce hydrophobic moieties into HA. In this reaction, the hydroxyl or carboxyl groups of HA can react with various alcohols to form ester bonds, thereby altering the hydrophobicity and improving the stability of HA-based biomaterials [25,29]. Chemical cross-linking is widely used to transform HA into hydrogel structures with improved stability. Cross-linked HA hydrogels exhibit superior mechanical properties and slower degradation compared with uncross-linked HA, which enhances their suitability as scaffolds for tissue engineering and regenerative medicine [27].

### 2.3. Biological Functions

HA is synthesized at the plasma membrane by hyaluronan synthases and directly extruded into the extracellular space, where it becomes a key component of the extracellular matrix and is widely distributed in connective tissues and biological fluids [30]. Furthermore, HA is a major glycosaminoglycan component of the endothelial glycocalyx, a carbohydrate-rich layer that covers the apical surface of vascular endothelial cells [31]. HA is involved in a wide range of biological processes, including wound healing, tissue repair and regeneration, extracellular matrix organization, angiogenesis, and regulation of cell migration. In addition, HA contributes to joint lubrication and exerts its biological functions through interactions with various cell-surface receptors [32].

The biological activity of HA is strongly dependent on its molecular weight and receptor interactions. High-molecular-weight HA (HMW-HA) is generally associated with tissue homeostasis, anti-inflammatory signaling, and protection of extracellular matrix integrity. In contrast, fragmented low-molecular-weight HA (LMW-HA), which is often generated during tissue injury or inflammation, can function as a danger-associated signal and stimulate inflammatory responses [16]. These distinct effects are mediated through different receptor systems. HA is not only a structural component of the extracellular matrix but also a dynamic signaling molecule that can differentially modulate wound healing, angiogenesis, immune responses, and tissue remodeling depending on its size and biological context [33].

Previous studies have reported a close association between HA and cancer progression. HA–receptor interactions have been shown to promote tumor growth, cell migration, and metastasis. In addition, the HA–hyaluronidase system has been implicated in the regulation of tumor angiogenesis [24]. In contrast, inhibition of HA synthesis using 4-methylumbelliferone (4-MU) has been reported to exert antitumor effects in various cancer models. Moreover, sulfated HA derivatives can inhibit hyaluronidase activity and suppress tumor progression [34]. Nevertheless, in cancer-related contexts, HA accumulation in the tumor microenvironment can promote cell motility, invasion, and angiogenesis through HA receptor-mediated signaling. Conversely, inhibition of HA synthesis or HA–receptor interactions has been shown to suppress tumor progression in certain models, although these effects remain context-dependent across different cancer types. Collectively, these findings underscore the dual and context-dependent roles of HA in tumor progression, acting as both a promoter and suppressor depending on molecular and microenvironmental conditions.

### 2.4. Degradation

HA degradation is mediated by a family of enzymes known as hyaluronidases. In humans, several hyaluronidase isoenzymes have been identified, including HYAL1–HYAL5 and HYALP1. These enzymes cleave the glycosidic bonds of HA, thereby reducing polymer length and generating lower-molecular-weight fragments. This process not only accelerates HA turnover but may also alter biological signaling by shifting HA from a homeostatic high-molecular-weight form to bioactive low-molecular-weight fragments. Consequently, hyaluronidases contribute not only to extracellular matrix remodeling but also to the regulation of the tumor microenvironment [23,35].

In addition to enzymatic degradation, HA can also undergo non-enzymatic cleavage through various physicochemical processes. Non-enzymatic degradation can be triggered by acidic or alkaline conditions, elevated temperature, mechanical stress, and reactive oxygen species (ROS) [29]. These factors promote hydrolysis or oxidative cleavage of the HA backbone, leading to reduced molecular weight, lower viscosity, weaker mechanical properties, and faster loss of scaffold integrity [26]. In tissue engineering applications, such degradation behavior is highly relevant because it determines the residence time, structural stability, and biological function of HA-based biomaterials in vivo [23]. For example, ultrasound-mediated degradation provides a convenient and controllable strategy to modulate the molecular weight and bioactivity of HA. Elvitigala et al. reported that sonication reduced the molecular weight of phenolated HA, decreased its viscosity and hydrogel stiffness, and significantly enhanced endothelial cell migration, proliferation, and capillary-like network formation in vitro. These effects were associated with increased CD44 and PI3K expression, suggesting that ultrasound-degraded HA may promote angiogenesis through CD44-mediated signaling. Therefore, ultrasonication represents a useful approach for tailoring HA-based biomaterials for vascularized tissue engineering [36].

## 3. Chemical Modification of HA

HA is frequently modified through chemical cross-linking to form hydrogels with tunable physicochemical properties. HA modification strategies are broadly classified into covalent crosslinking, physical self-assembly, and photo/enzymatic crosslinking approaches. Moreover, HA can be incorporated into natural or synthetic polymer systems to improve mechanical strength, biocompatibility, and biological functionality [37]. Carboxyl and hydroxyl groups are the two most chemically modified HA groups. The carboxyl groups on HA can be cross-linked by ester bonds, the hydroxyl groups can be cross-linked by ether bonds, or there are other chemical reactions. Furthermore, crosslinking reactions have been reported under basic, neutral and acidic conditions using carbodiimides, aldehydes, sulfides and multifunctional epoxides [38].

Esterified HA biomaterials were prepared by alkylation of HA with alkyl halides and ammonium salts in dimethylformamide solution. HA ester materials can be produced as fibers, films and sponges. The mechanical properties of esterified HA materials are strongly influenced by their degree of modification. Increasing the degree of esterification enhances the formation of hydrophobic domains within the polymer network, resulting in improved mechanical strength and structural stability. Moreover, the formation of a more compact polymer chain network reduces the susceptibility of HA derivatives to enzymatic degradation [25,39]. Due to their favorable biocompatibility and structural stability, esterified HA biomaterials have been developed into various scaffold forms, including meshes and porous sponges, to support fibroblast and chondrocyte growth and to facilitate cartilage and bone defect repair [40].

HA can also be modified with adipic, mono-, and polydihydrazides. Carbodiimide mediated coupling of ADH with the carboxyl group of HA forms HA-ADH, which permits further crosslinking and addition of polypeptides. To make a suitable material for tissue engineering applications, researchers produce hydrogels by further crosslinking. These materials may offer intrinsic biodegradation pathways and biological system recognition advantages [25].

Self-crosslinking represents an important strategy for modifying HA. In such systems, hydrophobic interactions and hydrogen bonding between carboxylate and acetamido groups can partially counteract the electrostatic repulsion between negatively charged HA chains. These short-range intermolecular interactions are favored when HA chains adopt an antiparallel arrangement, leading to the formation of reversible aggregates. The stability of these aggregates is influenced by environmental conditions such as temperature and ionic strength, as the interactions responsible for network formation are relatively weak. Despite this, self-crosslinked HA polymers have been widely explored for biomedical applications, including barriers to reduce postoperative adhesions and scaffolds for cell proliferation and tissue repair. Among these systems, HA benzoylcysteine derivatives represent a class of self-crosslinking materials capable of forming injectable hydrogels in situ without the need for toxic crosslinking agents. Such materials exhibit improved biocompatibility and structural stability, making them promising candidates for tissue engineering and regenerative medicine [41].

HA can also be cross-linked with multifunctional epoxides. To form ester and ether bonds, epoxy groups react with -COOH and -OH functional groups. Recent studies have shown that HA-agarose composites were prepared using epichlorohydrin as a cross-linking agent, and HA-collagen scaffolds were prepared using ethylene glycol diglycidyl ether [42,43]. Under acidic conditions, glutaraldehyde (GTA) can react with the functional groups of HA to form hemiacetal or ether linkages, resulting in cross-linked HA hydrogels. This crosslinking reaction generates a stable and water-insoluble polymer network [44].

HA can be chemically crosslinked using various reagents to enhance its structural stability and mechanical properties. One common approach involves the use of divinyl sulfone (DVS), which reacts with hydroxyl groups on the HA backbone to form ether linkages, resulting in a three-dimensional network of sulfonyl-bisethyl crosslinks. The crosslinking reaction typically occurs in alkaline aqueous conditions at room temperature, producing stable HA hydrogels. In addition, aldehyde-based crosslinking methods, such as reactions with formaldehyde, have also been employed to form crosslinked HA networks under neutral conditions through interactions with nucleophilic functional groups. The resulting HA hydrogels have been widely investigated for their physicochemical properties, including swelling behavior and degradation characteristics, as well as their suitability for cell culture and other biomedical applications [45].

Carbodiimide-mediated crosslinking is another common strategy for modifying HA-based biomaterials. In this approach, carbodiimide reagents first activate carboxyl groups on the polysaccharide to form reactive intermediates, which subsequently react with nucleophilic groups to generate stable amide bonds. To enhance the mechanical stability of composite scaffolds, HA–collagen matrices are often fabricated by freeze-drying and subsequently crosslinked using 1-ethyl-3-(3-dimethylaminopropyl)carbodiimide (EDC). These crosslinked scaffolds exhibit improved structural integrity and have been widely investigated for tissue engineering and cell culture applications [46,47].

Tyramine modification is an important and widely used strategy for HA functionalization because it introduces phenolic groups onto the HA backbone and enables mild crosslinking under biologically relevant conditions. In particular, tyramine-modified HA can form hydrogels through horseradish peroxidase (HRP)/H_2_O_2_-mediated gelation, an enzymatic system that allows injectable and in situ-forming hydrogels with tunable gelation kinetics and mechanical strength. Lee et al. showed that HA–tyramine hydrogels exhibit independently tunable gelation rate and stiffness by adjusting HRP and H_2_O_2_ concentrations, highlighting their utility in tissue engineering and drug delivery [48,49].

Photocrosslinking is another important HA modification strategy because it provides spatiotemporal control over hydrogel formation and matrix properties. Elvitigala et al. reported a visible-light-mediated system based on phenolated HA and phenolated gelatin, in which irradiation in the presence of tris(2,2′-bipyridyl)ruthenium(II) chloride and sodium persulfate simultaneously induced polymer crosslinking and oxidative HA degradation. By tuning the irradiation time, the authors were able to regulate HA molecular weight, hydrogel stiffness, and endothelial cell network formation, demonstrating that photocrosslinking can modulate both the physicochemical and biological properties of HA-based hydrogels [50].

Furthermore, HA has been oxidised by periodate to form hyaluronic dialdehyde (HDA), and methacrylated and photocrosslinked using Irgacure 2959 in N-vinyl pyrrolidone as an initiator to generate hydrogels and scaffold materials. The former one has the advantage of forming gels and scaffolds without cross-linking agents [51,52], while the latter has the potential to be used as soft tissue structures and materials. Representative methods, reagents, reaction conditions, advantages, and limitations are summarized in Table 1.

## 4. HA Cell Surface Receptors

The biological functions of HA are primarily mediated through its interactions with HA-binding proteins, collectively known as hyaladherins. These proteins include membrane-bound receptors such as CD44 and the receptor for hyaluronan-mediated motility (RHAMM, also known as CD168), as well as extracellular matrix components such as aggrecan and other proteoglycans [53]. The diversity of identified hyaladherins continues to expand as additional HA-binding proteins are discovered. Although the functions of many hyaladherins have been well characterized, the roles of several others remain incompletely understood.

Upon binding to cell-surface receptors such as CD44 and RHAMM, HA can activate a variety of intracellular signaling pathways that regulate cellular processes including differentiation, proliferation, migration, and HA turnover. These receptors act as key mediators of HA-dependent signaling, enabling cells to respond to changes in the extracellular environment [54,55].

CD44 is a multidomain transmembrane glycoprotein encoded by a highly conserved gene. Alternative splicing of CD44 transcripts generates multiple isoforms with distinct structural features and biological functions [56,57]. It is commonly seen in most human cell types. The affinity of CD44 for HA directly affects the action of the HA molecule. This affinity depends on the concentration and molecular weight of the molecule, phosphorylation of serine residues and/or glycosylation of the extracellular domain. Furthermore, CD44 can interact with a variety of ligands, including growth factors, extracellular matrix components, cytokines, and matrix metalloproteinases (MMPs). In addition, binding of high-molecular-weight HA (HMW-HA) can induce clustering of CD44 on the cell surface, which facilitates receptor activation and downstream signaling [57,58]. RHAMM, also known as CD168, is another HA receptor expressed in multiple cell types. RHAMM regulates cell migration through interactions with cytoskeletal proteins and plays important roles in tissue repair and inflammatory responses [59].

Stem cells derived from different sources are widely used as seed cells in tissue engineering. HA regulates stem cell behavior by interacting with cell-surface receptors such as CD44 and the receptor for hyaluronan-mediated motility (RHAMM). These interactions trigger intracellular signaling cascades that control stem cell proliferation, differentiation, and migration. Similar to CD44, RHAMM exists in several isoforms produced from highly conserved gene transcripts. The HA–RHAMM complex can activate Src-dependent signaling and focal adhesion kinase (FAK) pathways, which subsequently regulate cytoskeletal dynamics and cell motility [60]. This receptor is also associated with microtubules, mitochondria and nuclei, and the CD44 receptor, which means that it can be found both inside and outside cells [61]. Both CD44 and RHAMM are expressed in embryonic tissues and contribute to the migration of HA-rich cells during development. Nevertheless, knockout studies have shown that deletion of either receptor does not cause embryonic lethality, indicating functional redundancy and compensatory mechanisms among HA receptors [62]. This means that there may be other matrix proteins involved.

Lymphatic vessel endothelial hyaluronan receptor 1 (LYVE1) is a transmembrane glycoprotein that is somewhat similar with CD44. But unlike CD44, it is expressed by lymphatic endothelia [63]. Thus, it is used as a marker to differentiate between blood vessels and lymphatic vessels. LYVE1 is required for the transport of HA from tissue to lymphatic vessels by lymphatic endothelial cells, and indirect involvement of LYVE1 is also required for the mediated entry of leukocytes into lymphatic vessels [64].

## 5. Mechanistic Links Between HA Physicochemical Properties and Cellular Responses

Hyaluronic acid (HA) should not be regarded merely as a passive structural biomaterial, because its physicochemical properties actively shape the signaling environment perceived by cells [9,13]. Among these properties, molecular weight is a major determinant of HA bioactivity [9,16]. High-molecular-weight HA (HMW-HA) generally stabilizes tissue homeostasis, preserves extracellular matrix integrity, and suppresses inflammatory activation, whereas low-molecular-weight HA (LMW-HA) fragments generated during tissue injury or degradation more often function as danger-associated molecular cues that promote inflammatory signaling and matrix remodeling [16,23]. Mechanistically, these distinct effects are mediated through differential receptor engagement. HMW-HA can promote CD44 clustering and receptor-dependent homeostatic signaling, while fragmented HA is more likely to activate pro-inflammatory pathways through CD44, RHAMM, and innate immune receptors such as TLR2 and TLR4 [15,16]. Consequently, HA degradation does not simply reduce polymer size, but also converts the material from a matrix-preserving signal into a biologically active remodeling cue [16,23]. This concept is particularly important in tissue engineering, because scaffold degradation can directly change not only material stability but also the inflammatory and regenerative signaling milieu surrounding implanted cells [23,26].

In addition to molecular size, the mechanical features of HA-based biomaterials—including stiffness, viscoelasticity, and stress relaxation—strongly influence cell fate by regulating mechanotransduction [12,27]. Native HA is highly hydrated but mechanically weak, which is why crosslinking and chemical modification are commonly introduced to tune its structural stability and rheological behavior [9,27,51,52]. These changes alter how cells spread within the matrix, generate cytoskeletal tension, and form focal adhesions [12,60]. As a result, matrix mechanics can modulate downstream signaling pathways such as Src- and FAK-related cascades, which are closely linked to cell motility, proliferation, and lineage specification [60]. In tissue engineering settings, this has important implications for stem cell fate regulation: softer and more viscoelastic HA matrices may better preserve rounded cell morphology and support chondrogenic or other soft-tissue-associated phenotypes, whereas stiffer HA-based or composite matrices may enhance cell spreading and provide a more favorable microenvironment for osteogenic differentiation [12,51,52]. Thus, the biological performance of HA-based scaffolds depends not only on biochemical composition but also on how effectively the material transmits mechanical information to cells [12,27].

A further mechanistic advantage of HA lies in its role as an extracellular matrix-mimetic material [9,13]. Because HA possesses strong water-binding capacity and space-filling properties, HA-based hydrogels generate highly hydrated microenvironments that resemble many native soft tissues [9,26]. This hydrated matrix architecture facilitates nutrient diffusion, lubrication, and cell migration while reducing excessive cell–cell compression and helping maintain cell viability [9,26]. Importantly, this ECM-mimetic function is not static. As HA is enzymatically or oxidatively degraded, the matrix gradually loses both hydration-dependent mechanical buffering and structural integrity, while simultaneously releasing fragments with altered signaling functions [23,26]. Therefore, the biological effects of HA-based biomaterials should be understood as the integrated outcome of three interdependent variables: molecular weight-dependent receptor signaling, matrix mechanics-mediated mechanotransduction, and hydration-driven ECM mimicry [9,12,15,16,23,26]. This integrated framework more accurately explains why HA-based scaffolds can exert markedly different effects on inflammation, cell migration, differentiation, and tissue remodeling depending on formulation, crosslinking strategy, and degradation state, and provides a conceptual framework for linking material design parameters to biological outcomes in HA-based systems [12,15,16,23,27].

## 6. Natural Polymer Comparison in Tissue Engineering

Compared with other natural polymers commonly used in tissue engineering, HA exhibits several distinctive advantages [9,13,43]. Collagen is a major structural component of the native extracellular matrix and provides excellent intrinsic bioactivity and cell-adhesive properties, but its relatively rapid degradation and limited mechanical stability often restrict its independent use as a scaffold [40,43]. Alginate is attractive because of its low cost, mild gelation conditions, and good processability, especially in hydrogel and bioprinting applications; however, it lacks intrinsic mammalian cell-binding motifs and therefore often shows limited biological activity unless further modified [43]. Chitosan possesses favorable biocompatibility, biodegradability, and antimicrobial properties, making it particularly useful for wound healing and infection-related applications, although its poor solubility under physiological conditions and relatively weak mechanical performance can limit broader use [43]. In contrast, HA is a native, non-sulfated glycosaminoglycan of the mammalian extracellular matrix that combines high water-retention capacity, viscoelasticity, and receptor-mediated bioactivity [8,9,13]. Through interactions with receptors such as CD44 and RHAMM, HA can actively regulate cell migration, proliferation, inflammation, and tissue remodeling, making it especially suitable for constructing highly hydrated and cell-instructive microenvironments [8,9,13,15]. Nevertheless, similar to other natural polymers, HA also suffers from limited mechanical strength and rapid enzymatic degradation, and therefore is often chemically modified or combined with other biomaterials to improve its stability and functionality [8,9,13,43]. A brief comparison of HA with other commonly used natural polymers, including collagen, alginate, and chitosan, is provided in Table 2 to better contextualize the unique advantages and limitations of HA in tissue engineering.

## 7. Application of HA in Tissue Engineering

In tissue engineering applications, HA is widely used due to its ability to form three-dimensional, native ECM-like structures, making it an attractive scaffold material. HA hydrogels can mimic the water content of human tissues, support the presentation and retention of growth factors to promote tissue regeneration, and facilitate the exchange of metabolic wastes [74]. HA has also been reported to support tissue rejuvenation and repair processes, thereby enhancing the therapeutic performance of scaffold-based systems. This contributes to improved cell viability, adhesion, and overall tissue regeneration capacity [75]. In this review, the applications of HA in bone, cartilage, vascular, skin, and soft tissue engineering are discussed. Representative HA-based scaffolds and biomaterial platforms for different tissue engineering applications are summarized in Table 3, with emphasis on scaffold composition, relevant cell types, key regenerative outcomes, and current limitations.

### 7.1. Bone Tissue Engineering

HA has been widely studied and applied in bone tissue engineering, usually as a molecular carrier or scaffold to promote bone tissue regeneration. Previous reports indicate that it has been used in the field of cranial and alveolar bone [88,89]. However, the mechanical properties of HA are weaker than that of human bone. Therefore, a composite material with HA as a scaffold is a better choice.

HA can regulate bone formation and cell differentiation by interacting with CD44 and CD168 on cells. CD44 was originally identified as receptors for hyaluronan or HA and later to several other ligands including osteopontin (OPN), collagens, and matrix metalloproteinases [90,91].

Studies have shown that N-cadherin-modified HA can increase the potential of human mesenchymal cells to differentiate into bone. N-cadherin is a protein that plays an important role in mediating cell–cell interactions in bone formation. Some studies show that HA hydrogels are biofunctionalized with an N-cadherin mimetic peptide to mimic the pro-osteogenic niche in the endosteal space to promote the osteogenesis of human mesenchymal stem cells (hMSCs). Results show that the conjugation of the N-cadherin peptide in the HA hydrogels enhances the expression of the osteogenic marker genes in the seeded hMSCs. Furthermore, the biofunctionalized HA hydrogels promote the alkaline phosphatase activity, type I collagen deposition, and matrix mineralization by the seeded hMSCs under both in vitro and in vivo conditions [76].

**Figure 2:**
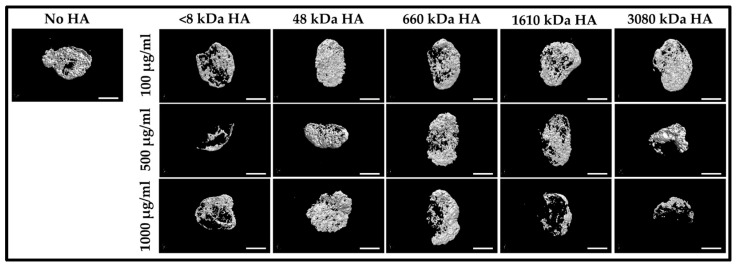
Effect of HA molecular weight on osteogenic activity. HA polymers with a molecular weight of 48 kDa exhibited the highest osteogenic potential when applied at a concentration of 100 μg/mL (dosage volume: 20 μL) in combination with BMP-2 (10 μg per sample; BMP-2 concentration: 1 μg/μL; solution volume: 10 μL/sample) in the experimental model [78]. Licensed under CC-BY-NC 4.0.

As a molecular carrier, HA can carry bone morphogenetic protein 2 (BMP-2) to increase bone formation, usually for fracture healing. BMP-2 has been certified for interbody fusion, open tibial fractures and alveolar bone expansion, and is a potent osteogenic molecule. In addition, BMP-2 significantly improved fusion rates, pseudarthrosis-related reoperations, and donor-site morbidity of iliac crest bone grafts. However, the value of BMP-2 is negatively affected because of its significant costs. As a result, further research investigating ways to minimize the costs associated with BMP-2 use can further improve its value in spine surgery. Previous studies have shown that thiolated-HA releases BMP-2 in low bursts and sustainably. Compared with collagen sponge, this HA-hydrogel can release more BMP-2 and induce bone formation. This highlights the therapeutic potential of hydrogels, particularly hyaluronan-based, for the delivery of BMP-2 for the treatment of bone defects and may help abrogate the adverse clinical effects associated with high dose growth factor use [77]. Another investigated whether HA enhances the osteogenic potential of BMP-2/ACS for bone regeneration. In a rat subcutaneous bone induction model, optimal HA size and concentration were identified by micro-CT, followed by histomorphometric evaluation of bone formation, construct volume, and vascular and macrophage densities. These findings indicate that HA can promote osteogenic and angiogenic activity of BMP-2/ACS, potentially allowing lower BMP-2 dosages and reducing associated side effects [78] (Figure 2).

### 7.2. Cartilage Tissue Engineering

HA–based materials are among the most widely investigated biomaterials in cartilage tissue engineering because they provide a favorable microenvironment for chondrocyte adhesion, proliferation, and extracellular matrix deposition. Moreover, HA-based scaffolds have also shown significant potential in osteochondral tissue engineering for the simultaneous regeneration of cartilage and subchondral bone [92].

HA promotes cartilage formation through several mechanisms. First, HA has the ability to induce stem cells to differentiate into chondrocytes [75]. Second, HA is involved in the maintenance of the chondrocyte phenotype [46]. Third, HA promotes the deposition of ECM that is critical for cartilage tissue engineering. In addition, the HA-containing hydrogel material has a lower oxygen concentration, which is favorable for the growth of chondrocytes [80]. HA–based hydrogels can also serve as cell carriers to encapsulate stem cells or chondrocytes and deliver them to defect sites. These injectable systems provide a supportive microenvironment that enables cells to proliferate, differentiate, and regenerate cartilage tissue in situ [93,94].

In recent years, methacrylated HA (MeHA or HAMA) hydrogels have attracted increasing attention because they can be rapidly gelled through simple photocrosslinking. This strategy enables the encapsulation of seed cells within the hydrogel network, providing a three-dimensional microenvironment that supports cell survival, proliferation, and tissue regeneration [52]. Previous studies demonstrated that both in vitro and in vivo culture of mesenchymal stem cell-loaded HA hydrogels allowed chondrogenesis. In situ crosslinking strategies, including thermal and photo-crosslinking methods, have emerged as promising approaches for hydrogel fabrication in tissue engineering. In particular, photo-crosslinked HA hydrogels have attracted increasing attention because of their excellent biocompatibility and tunable mechanical properties. As a natural component of cartilage extracellular matrix, HA can interact with mesenchymal stem cells (MSCs) through cell-surface receptors such as CD44, thereby regulating cell adhesion, proliferation, and differentiation. Consequently, photo-crosslinked HA hydrogels have been widely explored as scaffolds for promoting MSC-mediated cartilage regeneration [52,81].

**Figure 3:**
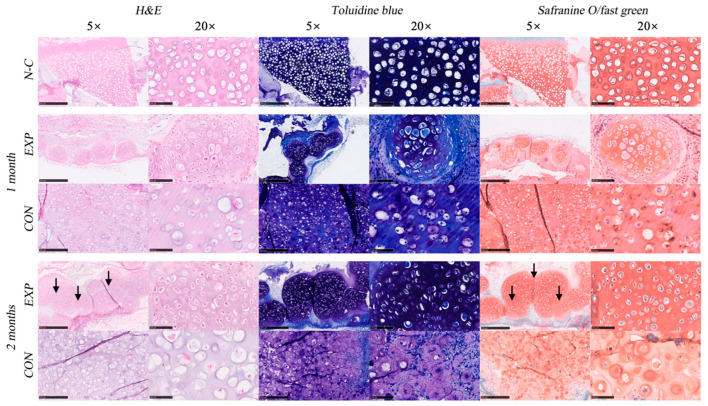
Histological evaluation of engineered cartilage constructs. Representative H&E staining, toluidine blue staining, and Safranin O/Fast Green staining of native cartilage (N-C), experimental group (EXP), and control groups (CON) at 1 and 2 months after implantation. Fusion and integration of adjacent cartilage spheroids are observed in the EXP group at 2 months, as indicated by black arrows [79]. Licensed under CC-BY-NC 4.0.

In addition, loading cartilage spheroids into HA methacrylate (HAMA) and gelatin methacrylate (GelMA) cross-linked hybrid hydrogels can improve cell proliferation and aggregation functions and inhibit dedifferentiation and hypertrophy to maintain chondrocyte phenotype. A hybrid gel composite of GelMA and HAMA can enhance the strength of materials while ensuring extracellular matrix formation and chondrocyte phenotype maintenance and was proven to be more conducive to chondrogenic differentiation [79] (Figure 3). The above-mentioned materials can be applied to nose and auricle reconstruction and plastic surgery, which require very high strength and shape of the materials.

### 7.3. Blood Vascular Tissue Engineering

HA is an important component of vascular tissue. Previous studies have shown that HA has the effect of promoting the migration and subsequent activation of tissue macrophages and neutrophils. When they are activated, angiogenesis can occur. Macrophages and neutrophils secrete angiogenic and mitogenic factors to form granulation tissue [46]. Furthermore, HA has been shown to have the ability to promote endothelialization and vascular replacement of vascular substitutes, and can directly induce complete angiogenesis. HA can be a useful temporary scaffold for guiding arterial and venous regeneration [84].

**Figure 4:**
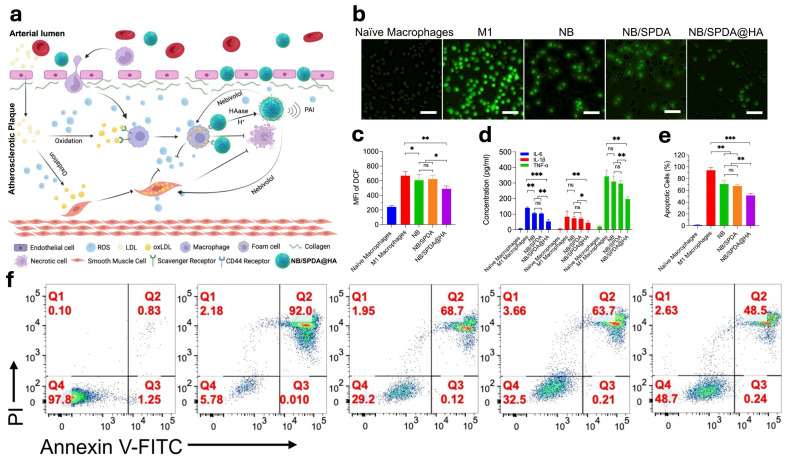
PAI-guided and CD44-targeted anti-ROS therapy for atherosclerosis using NB/SPDA@HA nanoparticles. (**a**) Schematic illustration of the PAI-guided and CD44-targeted anti-ROS therapy for atherosclerosis. NB/SPDA@HA nanoparticles release the loaded drug Nebivolol (NB) in response to the plaque microenvironment, including acidic pH and elevated hyaluronidase levels. The released NB locally reduces reactive oxygen species (ROS) and inflammation within atherosclerotic plaques. In addition, photoacoustic imaging (PAI) enables real-time monitoring and precise localization of the therapeutic process. This targeted strategy aims to both visualize and attenuate the progression of atherosclerosis in a plaque-specific manner. Anti-inflammatory effects of NB/SPDA@HA nanoparticles. Green fluorescence indicates ROS generation. (**b**) Representative fluorescence images of DCF staining for intracellular ROS detection. (**c**) Flow cytometry quantification of ROS levels using DCF fluorescence after different treatments. (**d**) Quantification of inflammatory cytokine secretion from RAW264.7 cells. (**e**) Flow cytometry analysis of apoptosis in RAW264.7 cells. (**f**) Representative FACS plots of apoptosis analysis in RAW264.7 cells MFI, mean fluorescence intensity; ns, not significant; * *p* < 0.05; ** *p* < 0.01; *** *p* < 0.001 [86]. Licensed under CC-BY-NC 3.0.

HA is a modulator of atherosclerotic lesion stability [95]. It is highly upregulated at atheroprone regions in mouse models of atherosclerosis and may regulate vascular smooth muscle cell (VSMC) migration, proliferation, leukocyte activation, and lipid accumulation within the plaque to alter stability [1,96]. However, the receptor CD44 also plays a critical role in atherogenesis. CD44 is a type 1 transmembrane receptor and its standard form is widely expressed in multiple cell types. Under inflammatory conditions, CD44 is upregulated and functionally activated on vascular endothelial, smooth muscle and inflammatory cells. Therefore, HA is an attractive therapeutic target to halt the course of atherosclerosis. Atherosclerosis remains a leading cause of cardiovascular disease, yet plaque-targeted pharmacotherapy is still lacking. Nebivolol (NB), a third-generation β-blocker, exhibits potent antioxidative activity by inhibiting and scavenging ROS. Here, they developed a macrophage-targeting nanotheranostic system for atherosclerotic plaques. Mesoporous silica nanoparticles (MSNs) were coated with polydopamine (PDA) for photoacoustic imaging (PAI) and conjugated with HA to target CD44-overexpressing macrophages. NB-loaded NB/SPDA@HA nanoparticles reduced ROS levels and inflammation, inhibited apoptosis and foam cell formation, and enabled accelerated drug release in the acidic and hyaluronidase-rich plaque microenvironment. This dual-responsive platform provides a promising strategy for targeted imaging and therapy of atherosclerosis [86] (Figure 4).

When HA is applied to a vascular substitute, it should be blood compatible, absorbable and permanent. Chitosan–HA-based polyelectrolyte membranes exhibit good biocompatibility and have potential as vascular substitutes [82]. In addition, previous studies showed that the nanofibrous structures prepared by HA had the ability to promote endothelial cell proliferation, and no hemolysis and coagulation were detected in the scaffolds [83]. Furthermore, HA is also used in tissue ischemia treatment and tissue replacement, which can promote tissue vascularization and microvascular formation. Gelatin-based hydrogels with low-molecular-weight HA derivatives promote endothelial cell motility in vitro, and their desirable mechanical properties contribute to the construction of vascularized dense tissue [85].

### 7.4. Skin and Soft Tissue Engineering

Skin substitutes represent one of the most widely studied and successfully applied products in tissue engineering. A common strategy involves seeding keratinocytes and fibroblasts into biomaterial matrices to construct engineered skin equivalents. However, the stability of these constructs is often limited, with the functional skin equivalent typically maintained for only about eight weeks. One major limitation is the excessive contraction of the ECM, which may impair the formation of a physiologically relevant tissue microenvironment. The ECM normally consists of a complex network of structural proteins and polysaccharides, including fibrin, lipids, polysaccharides, and glycosaminoglycans, which collectively maintain tissue architecture and regulate cellular behavior. To overcome these limitations, HA–based biomaterials have been explored as alternative scaffolds [97]. Previous studies have demonstrated that keratinocytes cultured on esterified HA scaffolds exhibit morphological and functional characteristics resembling native dermal tissue, enabling the formation of skin equivalents with improved stability and extended maintenance periods [32,87]. In addition to skin tissue engineering, HA has also been investigated for improving adipose tissue retention. Experimental studies using nude mouse models have shown that HA can enhance the early survival of fat grafts and prolong graft volume maintenance [98].

**Figure 5:**
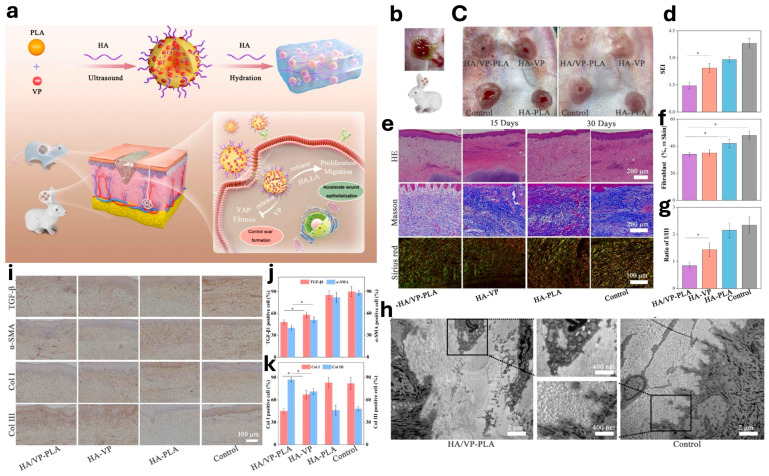
HA/VP-PLA nanogel system for scarless wound healing. (**a**) Schematic illustration of HA/VP-PLA promoting scarless wound healing by accelerating wound re-epithelialization and regulating scar formation. (**b**) Schematic representation of the application of HA/VP-PLA in a rabbit wound model. (**c**) Representative images of wound tissue at 15 and 30 days post-surgery. (**d**) Quantification of the scar elevation index (SEI) of tissues. (**e**) Histological analysis of tissues using H&E, Masson’s trichrome, and Sirius red staining. (**f**) Quantification of fibroblast proliferation. (**g**) Statistical analysis of the collagen I to collagen III ratio. In panels (**d**,**f**,**g**), the purple groups represent HA/VP-PLA, the orange groups represent HA-VP, the blue groups represent HA-PLA, and the gray groups represent the control groups (*: *p* < 0.05). (**h**) Ultrastructural differences between HA/VP-PLA-treated and control tissues. (**i**) Immunohistochemical analysis of fibrosis-related biomarkers. (**j**) Quantitative analysis of TGF-β1 and α-SMA expression. (**k**) Quantitative analysis of collagen I and collagen III expression In panels (**j**) and (**f**), The red and blue groups represent collagen I and collagen III, respectively (*: *p* < 0.05) [99]. Licensed under CC-BY-NC 4.0.

Additionally, the intrinsic properties of HA (such as biocompatibility, biodegradability, and hydrophilic character), have been used to produce different wound dressings, namely sponges, films, hydrogels, and electrospun membranes [18]. The functions of wound dressings are mostly to promote cell migration, avoid tissue dehydration and support the healing process. HA is usually combined with other polymers or produced through chemical synthesis to produce HA derivatives. To illustrate: mixing HA with CS and ALG, using the freeze-drying technique to generate porous sponges, incorporating HA into bioactive molecules (such as growth factors, natural product extracts, and sulfadiazine (SD)) and inorganic compounds to produce a thin film as a result. The EDC coupling was functionalized with bisphosphate (BP) side groups, and then silver ions were added to the BP-modified HA hydrogel. The in vitro assays showed that the produced hydrogels were able to inhibit the growth *S. aureus* and *E. coli*. Furthermore, the in vivo assays demonstrated that the animals treated with the hydrogel showed a smaller wound area in comparison to the untreated group, 6 days after the wound was induced. A blend of HA, collagen (COL), and poly(L-lactide-co-ε-caprolactone) (PLC) was prepared to produce a nanofibrous membrane able to support cell proliferation and promote the vascularization process. The characterization of morphological properties and swelling profile of the produced membrane demonstrated that the PLC/COL/HA fibers presented lower diameter values (569 ± 188 nm) and a higher water uptake capacity (103 ± 13%), in comparison to the PLC membrane (which presented mean diameter values of 641 ± 104 nm and 66 ± 4% of swelling ability) [18]. Delayed healing and excessive scar formation remain major challenges in skin and soft tissue engineering. To address these issues, HA-based nanomaterials have recently been developed to improve wound repair outcomes. As shown in Figure 5, HA-modified and verteporfin-loaded polylactic acid nanogels (HA/VP-PLA) represent a promising strategy for scarless wound healing. In this system, the HA coating facilitates fibroblast-targeted delivery, while the release of HA and lactic acid promotes wound re-epithelialization and tissue remodeling (Figure 5a). In a rabbit wound model, HA/VP-PLA treatment improved gross wound closure, reduced the scar elevation index, and resulted in more organized tissue architecture compared with the control groups (Figure 5b,c). As shown in Figure 5d, HA/VP-PLA significantly reduced the scar elevation index and residual wound area compared with the other treatment groups, indicating improved wound closure and attenuated scar formation. In Figure 5e, H&E and Masson’s trichrome staining revealed better re-epithelialization, more complete tissue regeneration, and more organized collagen deposition in the HA/VP-PLA-treated wounds. In Figure 5f, quantitative analysis further demonstrated enhanced fibroblast proliferation in the HA/VP-PLA group, suggesting that this nanogel system promoted the cellular activity required for effective tissue repair. Consistently, Figure 5g showed a more favorable collagen I/collagen III ratio in HA/VP-PLA-treated wounds, indicating improved extracellular matrix remodeling and reduced fibrotic scar formation. The ultrastructural analysis in Figure 5h showed that the HA/VP-PLA group exhibited a more regular and compact extracellular matrix organization, whereas the control tissue displayed disordered fibrotic architecture. Immunohistochemical staining in Figure 5i revealed lower expression of fibrosis-related biomarkers in the HA/VP-PLA group than in the control groups. This finding was supported by the quantitative analyses in Figure 5j,k, which demonstrated significant downregulation of TGF-β1 and α-SMA, together with modulation of collagen I and collagen III expression. Collectively, these results suggest that HA/VP-PLA not only accelerates wound healing but also regulates fibroblast activity and collagen remodeling to promote scarless repair [99].

### 7.5. Bioprinting of HA-Based Bioinks

Three-dimensional (3D) bioprinting has emerged as a major strategy in tissue engineering because it enables the spatially controlled deposition of cells, biomaterials, and bioactive molecules to fabricate constructs with tissue-mimetic architecture [1,2]. Among the various biomaterials used for bioink formulation, HA has attracted increasing attention because it is a native component of the extracellular matrix and provides highly hydrated, viscoelastic microenvironments that are favorable for cell survival and tissue regeneration [1,8,51]. In addition, the biological activity of HA, including its interactions with receptors such as CD44 and RHAMM, makes HA-based bioinks more cell-instructive than many other hydrogel systems [8,51].

However, native HA alone is generally not ideal for direct bioprinting because of its limited mechanical strength, rapid degradation, and insufficient structural fidelity after extrusion or deposition [1,51]. To overcome these limitations, HA is frequently chemically modified to introduce photocrosslinkable or chemically reactive groups, thereby improving printability, gelation behavior, and post-printing stability [51,52]. Among these derivatives, hyaluronic acid methacrylate (HAMA or MeHA) is one of the most widely used HA-based bioinks. Methacrylation allows rapid photocrosslinking under mild conditions, enabling the fabrication of cell-laden constructs with tunable mechanical properties and relatively high shape fidelity [1,52]. This feature is particularly advantageous in cartilage and osteochondral tissue engineering, where bioprinted scaffolds must maintain defined geometries while supporting chondrogenic differentiation and extracellular matrix deposition [1,52].

The performance of HA-based bioinks is strongly influenced by rheological parameters such as viscosity, shear-thinning behavior, yield stress, and crosslinking kinetics [1,2]. For extrusion-based bioprinting, a suitable HA bioink should exhibit sufficient viscosity to maintain structural integrity after deposition, while also showing shear-thinning behavior to reduce cell damage during printing [1,2]. In practice, HA is often blended with other biomaterials, such as gelatin, GelMA, collagen, alginate, or decellularized extracellular matrix, to achieve a better balance between printability, cytocompatibility, and mechanical stability [1,43,51]. Such hybrid bioinks can also provide additional biochemical cues and improve the maturation of engineered tissues.

Recent studies have demonstrated that HA-based bioinks are particularly promising for the fabrication of cartilage, bone, skin, and vascularized soft tissue constructs [1,52]. In cartilage tissue engineering, HA-containing bioinks can mimic the highly hydrated native cartilage microenvironment and support chondrocyte phenotype maintenance and mesenchymal stem cell chondrogenesis [52]. In skin and soft tissue engineering, HA-based bioinks can enhance cell viability and matrix deposition while facilitating the fabrication of multilayered or irregularly shaped constructs [1,51]. Furthermore, because HA is readily modified and combined with functional molecules, HA-based bioinks also provide opportunities for integrating growth factors, nanoparticles, extracellular vesicles, and other bioactive agents into printed scaffolds to further enhance regenerative outcomes [8,51].

Despite these advantages, several challenges remain for the broader application of HA-based bioinks in 3D bioprinting. These include the relatively weak mechanical properties of pure HA hydrogels, limited long-term stability in vivo, and the need to optimize crosslinking conditions to preserve both print fidelity and cell viability [1,2,52]. In addition, the relationship between HA molecular weight, degree of chemical modification, rheological behavior, and biological performance still requires further systematic investigation [1,51,52]. Future advances in HA-based bioprinting are therefore expected to focus on multifunctional composite bioinks, dynamic crosslinking chemistries, and biofabrication strategies that more closely recapitulate the structural and biochemical complexity of native tissues, thereby highlighting the potential of HA-based bioinks as key platforms for engineering structurally and functionally complex tissues [1,51].

## 8. Discussion

Despite the promising applications of HA in tissue engineering and regenerative medicine, several challenges remain that may limit its broader clinical translation. One important issue is the molecular weight–dependent biological activity of HA, which remains controversial. HMW-HA generally exhibits anti-inflammatory and tissue-protective effects, whereas LMW-HA fragments can stimulate immune activation and promote inflammatory signaling through receptors such as CD44, TLR2, and TLR4 [16,18]. This size-dependent behavior may lead to inconsistent biological responses in different experimental or clinical settings. Another challenge is the immunomodulatory paradox of HA, in which HA can exert both anti-inflammatory and pro-inflammatory effects depending on its molecular size, concentration, and degradation state. For example, HMW-HA can suppress inflammatory responses through CD44-mediated signaling, whereas fragmented HA generated during tissue injury can activate macrophages and stimulate pro-inflammatory cytokine production [100]. This dual role complicates the design of HA-based biomaterials for therapeutic applications. Finally, the clinical translation of HA-based biomaterials remains limited by several practical considerations, including rapid enzymatic degradation by hyaluronidases, insufficient mechanical strength for load-bearing tissues, and challenges in large-scale manufacturing and regulatory approval [99,101]. These issues highlight the need for improved crosslinking strategies, composite biomaterials, and advanced biofabrication techniques such as 3D bioprinting to enhance the stability and functionality of HA-based scaffolds [33].

Future research on HA-based biomaterials is expected to focus on several emerging directions. The integration of HA with advanced biofabrication technologies such as 3D bioprinting offers significant opportunities for constructing biomimetic scaffolds with precise structural control and spatial distribution of cells and biomolecules. HA-based bioinks have shown promising potential for fabricating complex tissue constructs, including cartilage, skin, and vascular tissues [102,103]. The development of HA-based nanomedicine platforms has attracted increasing attention. Due to its excellent biocompatibility and ability to interact with HA receptors, HA can be utilized to construct nanoparticles or nanogels for drug delivery, gene therapy, and imaging applications [15]. These systems can improve drug stability, prolong circulation time, and enhance therapeutic efficacy. CD44-targeted delivery strategies represent an important therapeutic approach. CD44 is widely overexpressed in various pathological conditions, including cancer and inflammatory diseases. HA-modified nanoparticles can selectively bind to CD44-expressing cells, enabling targeted drug delivery and improved therapeutic outcomes. Collectively, the integration of HA with advanced biofabrication, nanotechnology, and receptor-targeted strategies may further expand the biomedical applications of HA in regenerative medicine and precision therapy [8].

## 9. Conclusions

Hyaluronic acid has emerged as a highly versatile biomaterial for tissue engineering and regenerative medicine because it combines native extracellular matrix relevance with excellent biocompatibility, hydrophilicity, viscoelasticity, and receptor-mediated bioactivity. Beyond serving as a structural polysaccharide, HA actively regulates cell adhesion, migration, proliferation, inflammation, and tissue remodeling through interactions with receptors such as CD44 and RHAMM, with its biological functions strongly influenced by molecular weight, degradation state, and matrix context. These characteristics distinguish HA from many other natural polymers and make it particularly valuable for constructing hydrated and cell-instructive biomaterial systems.

Recent advances in chemical modification and crosslinking strategies have greatly expanded the utility of HA-based biomaterials. Through esterification, carbodiimide-mediated coupling, tyramine modification, enzymatic gelation, aldehyde- and epoxide-based crosslinking, oxidation, and photocrosslinking, HA can be engineered into hydrogels, nanofibers, and composite scaffolds with tunable mechanical properties, degradation profiles, and biological performance. These developments have enabled broad applications of HA in bone, cartilage, vascular, skin, and soft tissue engineering, and have further promoted its integration into emerging fields such as 3D bioprinting, nanomedicine, and receptor-targeted therapeutic delivery.

Importantly, this review highlights that the performance of HA-based biomaterials cannot be fully understood solely from composition or application alone. Rather, their biological effects arise from the interplay among molecular weight-dependent signaling, matrix mechanics, receptor interactions, and ECM-mimetic hydration behavior. This integrated mechanistic perspective provides a framework for linking material design parameters to biological outcomes and helps explain why different HA formulations can produce markedly different cellular and regenerative responses.

Despite these advances, several important challenges remain, including limited mechanical strength, rapid enzymatic degradation, context-dependent immunomodulatory effects, and barriers to large-scale clinical translation. Future progress will likely depend on the development of multifunctional HA composites, improved control of degradation and mechanical cues, deeper understanding of receptor-mediated signaling, and the integration of advanced fabrication approaches such as 3D bioprinting. With these advances, HA-based biomaterials are expected to play an increasingly important role in tissue repair, disease modeling, and precision therapeutic applications, collectively positioning them as a versatile and evolving platform for next-generation regenerative medicine.

## Figures and Tables

**Table 1:** Hyaluronic acid crosslinking methods: representative reagents, reaction conditions, advantages and limitations.

Crosslinking Method	Representative Reagents	Typical Reaction Conditions	Advantages	Limitations	Ref.
Esterification	Alkyl halides, ammonium salts	Typically performed in DMF or other organic solvents through reaction of HA hydroxyl/carboxyl groups	Introduces hydrophobicity; improves structural stability; reduces enzymatic susceptibility; suitable for fibers, films, and sponges	May reduce hydrophilicity and native bioactivity; organic solvent use may limit biocompatibility/process simplicity	[25,39]
Carbodiimide-mediated crosslinking	EDC; often combined with collagen matrices	Mild aqueous conditions; carboxyl activation followed by reaction with nucleophiles to form amide bonds	Improves scaffold integrity; widely used for composite scaffolds; relatively versatile	Crosslinking efficiency depends on nucleophile availability; side reactions may occur	[46,47]
ADH-modified HA (HA-ADH)	Adipic dihydrazide (ADH) + carbodiimide coupling	Carboxyl activation followed by ADH grafting; used as precursor for further crosslinking	Provides reactive intermediate for further conjugation; useful for hydrogel fabrication	Requires extra synthetic step; final properties depend on degree of substitution and secondary crosslinking	[25]
Self-crosslinking/physical self-assembly	HA benzoylcysteine derivatives; no external crosslinker	Driven by hydrophobic interactions and hydrogen bonding; influenced by temperature and ionic strength	Avoids toxic crosslinkers; injectable; good biocompatibility; in situ gelation possible	Weaker intermolecular interactions; lower long-term mechanical stability	[41]
Epoxide crosslinking	Epichlorohydrin; ethylene glycol diglycidyl ether (EGDE)	Epoxy groups react with –COOH and –OH groups to form ester/ether linkages, often under controlled alkaline conditions	Forms stable covalent networks; improves structural robustness	Epoxide reagents may raise cytotoxicity concerns if residual reagent remains	[43]
Aldehyde crosslinking	Glutaraldehyde (GTA); formaldehyde	GTA often under acidic conditions; aldehydes react with nucleophilic groups to form crosslinked networks	Strong network formation; water-insoluble and structurally stable hydrogels	Residual aldehydes may be cytotoxic; purification is essential	[44,45]
DVS crosslinking	Divinyl sulfone (DVS)	Typically carried out in alkaline aqueous solution at room temperature; reacts mainly with hydroxyl groups to form ether linkages	Produces stable HA hydrogels; relatively simple chemistry; improves physicochemical stability	DVS is highly reactive and may present toxicity concerns; careful pH control and purification required	[45]
Oxidized HA-based crosslinking	Periodate oxidation to form hyaluronic dialdehyde (HDA)	Oxidation generates aldehyde groups, followed by spontaneous or secondary crosslinking	Can form gels/scaffolds without additional crosslinker in some systems; provides versatile reactive handles	Oxidation may alter backbone integrity and weaken mechanical properties if overdone	[51]
Methacrylation + photocrosslinking	Methacrylated HA (MeHA/HAMA), Irgacure 2959, N-vinyl pyrrolidone	Rapid photocuring after methacrylation under UV/light exposure	Fast gelation; tunable mechanics; suitable for cell encapsulation, bioprinting, and in situ gelation	Light exposure and photoinitiators may affect cell viability; properties depend strongly on degree of methacrylation	[51,52]
Visible-light photocrosslinking	HA-Ph, Gelatin-Ph, [Ru(bpy)_3_]^2+^, SPS	Visible-light irradiation at 450 nm induces phenol crosslinking and oxidative HA degradation in aqueous solution	Tunable stiffness and degradation; cytocompatible visible-light system; useful for regulating endothelial cell behavior and vascular network formation	Simultaneous degradation may reduce structural stability if overexposed; requires an additional adhesive component such as gelatin	[50]

**Table 2:** Brief comparison of hyaluronic acid with other natural polymers commonly used in tissue engineering.

Natural Polymer	Main Advantages in Tissue Engineering	Main Limitations	Distinctive Comparison with HA	Ref.
Collagen	Major structural component of native ECM; inherently bioactive; supports cell adhesion, cell–cell interaction, and tissue-like remodeling.	Poor mechanical strength, relatively rapid in vivo degradation, and relatively high production/isolation cost.	Collagen is generally superior to HA for direct cell adhesion and ECM mimicry, but HA offers stronger hydration, viscoelasticity, and receptor-mediated signaling. HA is also often used with collagen to compensate for collagen’s rapid degradation and to add biological tuning.	[65,66,67]
Alginate	Biocompatible, non-toxic, low-cost, easy to process, and readily gelled under mild ionic conditions; widely used as injectable hydrogels and bioinks.	Poor mammalian cell adhesion, weak/unstable mechanics in many settings, ion-exchange-driven dissolution, and limited enzymatic degradability in mammals unless chemically modified.	Alginate is often easier than HA for mild gelation and 3D bioprinting, but HA is biologically more instructive because it is a native mammalian ECM glycosaminoglycan that can regulate cells through receptors such as CD44 and RHAMM.	[8,68,69]
Chitosan	Biocompatible, biodegradable, bioadhesive, antimicrobial, and readily combined with other bioactive materials; attractive for wound healing and infected tissue repair.	Poor solubility at neutral/physiological pH, and pure chitosan scaffolds often show poor mechanical properties, shrinkage, and deformation.	Chitosan is particularly attractive when antibacterial activity, hemostasis, or adhesion is desired, whereas HA is more distinctive for hydration, viscoelasticity, and cell-instructive receptor signaling in native-like tissue microenvironments.	[70,71,72]
Hyaluronic acid	Native, non-sulfated glycosaminoglycan of mammalian ECM; high water retention, viscoelasticity, and lubricity; can modulate cell behavior through CD44/RHAMM and related HA receptors; widely used in hydrogels, coatings, and wound-healing systems.	Pure HA typically has limited mechanical strength and can undergo relatively rapid enzymatic degradation, so chemical modification or blending is often needed for load-bearing or long-term scaffold use.	Compared with collagen, alginate, and chitosan, HA’s most unique advantage is that it combines native ECM relevance with high hydration/viscoelasticity and receptor-mediated bioactivity, making it especially valuable for cell-instructive and immunomodulatory biomaterials.	[8,67,73]

**Table 3:** Representative HA-based scaffolds classified by tissue type: composition, cell type, key findings, limitations, and references.

Tissue Type	Scaffold/Composition	Cell Type	Key Findings	Limitations	Ref.
Bone	N-cadherin-functionalized HA hydrogel	hMSCs	Mimicked the osteogenic niche and enhanced osteogenic marker expression, alkaline phosphatase activity, type I collagen deposition, and matrix mineralization	HA alone has limited mechanical strength for load-bearing bone repair; biofunctionalization is often required to enhance osteogenesis	[76]
Bone	Thiolated HA hydrogel loaded with BMP-2	Not primarily cell-laden; growth factor delivery platform	Provided sustained BMP-2 release and promoted bone formation more effectively than collagen sponge in some settings	Growth factor-based systems may increase cost; osteogenic efficacy depends on release kinetics and dose control	[77]
Bone	HA + absorbable collagen sponge (ACS) + BMP-2	Host cells recruited in vivo	HA promoted BMP-2-induced osteogenesis and angiogenesis; specific HA molecular weight/concentration improved bone induction and may reduce required BMP-2 dose	Outcome depends strongly on HA size and concentration; still limited by the need for exogenous BMP-2	[78]
Cartilage/osteochondral	HA-based hydrogels	Chondrocytes, MSCs	Provide a favorable microenvironment for chondrocyte adhesion, proliferation, extracellular matrix deposition, and osteochondral regeneration	Pure HA hydrogels often lack sufficient mechanical strength and may require hybridization or crosslinking optimization	[79]
Cartilage	HA-containing hydrogel	Human adipose-derived MSCs and human chondrocytes co-culture	Facilitated chondrogenesis and matrix deposition; HA-supported low-oxygen-like microenvironment favorable for cartilage formation	Chondrogenic outcome depends on culture conditions and scaffold formulation	[80]
Cartilage	Photo-crosslinked HA hydrogel/HAMA-based hydrogel	MSCs or encapsulated seed cells	Rapid gelation, good biocompatibility, and tunable mechanics; supports cell encapsulation and cartilage regeneration	Photoinitiators and light exposure may affect cell viability; scaffold performance depends on degree of methacrylation/crosslinking	[52,81]
Cartilage	GelMA/HAMA hybrid hydrogel loaded with chondrocyte spheroids	Chondrocyte spheroids	Improved proliferation, spheroid integration, ECM formation, and maintenance of chondrocyte phenotype while reducing dedifferentiation and hypertrophy	More complex fabrication; hybrid systems require balancing printability, stiffness, and biological performance	[79]
Vascular	Chitosan–HA polyelectrolyte membrane	Wharton’s jelly derived stem cells/vascular-related cells	Showed good biocompatibility and potential use as vascular substitutes	Long-term hemocompatibility and in vivo remodeling remain concerns	[82]
Vascular	HA oligosaccharide-modified collagen nanofibers	Endothelial cells	Promoted endothelial cell proliferation and showed no evident hemolysis or coagulation, supporting potential vascular graft application	Nanofibrous scaffolds still require further validation for long-term vascular replacement	[83]
Vascular/ischemic tissue repair	Gelatin-based hydrogel with low-molecular-weight HA derivatives	Endothelial cells	Promoted endothelial motility and supported construction of vascularized dense tissue	Mechanical and long-term in vivo stability may require further improvement	[84,85]
Vascular disease targeting	HA-conjugated polydopamine-coated mesoporous silica nanoparticles (NB/SPDA@HA)	CD44-overexpressing macrophages	Enabled CD44-targeted delivery, reduced ROS and inflammation, inhibited apoptosis and foam cell formation, and supported plaque-targeted theranostics	Primarily a nanotherapeutic system rather than a bulk scaffold; translation requires safety and manufacturing optimization	[86]
Skin/soft tissue	Esterified HA scaffold	Keratinocytes	Supported formation of skin equivalents with improved stability and morphology resembling native dermal tissue	Functional skin substitutes remain limited by contraction and long-term stability	[32,87]
Skin/wound healing	HA-based wound dressings (sponges, films, hydrogels, electrospun membranes)	Fibroblasts, keratinocytes, host wound cells	Promote cell migration, prevent dehydration, support healing, and can be combined with bioactive molecules or antimicrobials	Mechanical strength and residence time vary widely among formats; often require combination with other polymers	[18]
Skin/wound healing	HA/collagen/poly(L-lactide-co-ε-caprolactone) nanofibrous membrane	Skin cells/host wound cells	Supported cell proliferation, enhanced water uptake, and promoted vascularization-related healing responses	Multicomponent fabrication may increase complexity; in vivo long-term outcomes require further validation	[18]

## Data Availability

No new data were created or analyzed in this study. Data sharing is not applicable to this article.

## Abbreviations

Hyaluronic acid	HA
Extracellular matrices	ECMs
Poly(N-isopropylacrylamide)	PNIPAM
Poly(ethylene glycol)	PEG
HA synthase	HAS
ATP-binding cassette	ABC
D-glucuronic acid	GlcA
N-acetyl-D-glucosamine	GlcNAc
High-molecular-weight HA	HMW-HA
Low-molecular-weight HA	LMW-HA
4-Methylumbelliferone	4-MU
Reactive oxygen species	ROS
Glutaraldehyde	GTA
Divinyl sulfone	DVS
1-Ethyl-3-(3-dimethylaminopropyl) carbodiimide	EDC
Hyaluronic dialdehyde	HDA
Adipic dihydrazide	ADH
Ethylene glycol diglycidyl ether	EGDE
Methacrylated HA	MeHA
Receptor for hyaluronan-mediated motility	RHAMM
Matrix metalloproteinases	MMPs
Focal adhesion kinase	FAK
Lymphatic vessel endothelial hyaluronan receptor 1	LYVE1
Osteopontin	OPN
Human mesenchymal stem cells	hMSCs
Bone morphogenetic protein 2	BMP-2
HA methacrylate	HAMA
Gelatin methacrylate	GelMA
Experimental group	EXP
Control groups	CON
Vascular smooth muscle cell	VSMC
Nebivolol	NB
Mesoporous silica nanoparticles	MSNs
Polydopamine	PDA
Photoacoustic imaging	PAI
Sulfadiazine	SD
Bisphosphate	BP
Poly(L-lactide-co-ε-caprolactone)	PLC
Verteporfin	VP
Scar elevation index	SEI
Absorbable collagen sponge	ACS
Three-dimensional	3D
Collagen	COL

## References

[1] Ding Y.-W., Zhang X.-W., Mi C.-H., Qi X.-Y., Zhou J., Wei D.-X. (2023). Recent advances in hyaluronic acid-based hydrogels for 3D bioprinting in tissue engineering applications. Smart Mater. Med..

[2] Khan M.U.A., Stojanovic G.M., Abdullah M.F.B., Dolatshahi-Pirouz A., Marei H.E., Ashammakhi N., Hasan A. (2024). Fundamental properties of smart hydrogels for tissue engineering applications: A review. Int. J. Biol. Macromol..

[3] Naegeli K.M., Kural M.H., Li Y., Wang J., Hugentobler E.A., Niklason L.E. (2022). Bioengineering Human Tissues and the Future of Vascular Replacement. Circ. Res..

[4] Zhang Y., Zhang C., Li Y., Zhou L., Dan N., Min J., Chen Y., Wang Y. (2023). Evolution of biomimetic ECM scaffolds from decellularized tissue matrix for tissue engineering: A comprehensive review. Int. J. Biol. Macromol..

[5] Ye B., Wu B., Su Y., Sun T., Guo X. (2022). Recent Advances in the Application of Natural and Synthetic Polymer-Based Scaffolds in Musculoskeletal Regeneration. Polymers.

[6] Ding Y., Duan Y., Yang F., Xiong Y., Guo S. (2023). High-transmittance pNIPAm gel smart windows with lower response temperature and stronger solar regulation. Chem. Eng. J..

[7] Xiao Z., Zhao S., Zhang X., Wei G., Su Z. (2022). Recent advances in peptide engineering of PEG hydrogels: Strategies, functional regulation, and biomedical applications. Macromol. Mater. Eng..

[8] Yasin A., Ren Y., Li J., Sheng Y., Cao C., Zhang K. (2022). Advances in Hyaluronic Acid for Biomedical Applications. Front. Bioeng. Biotechnol..

[9] Iaconisi G.N., Lunetti P., Gallo N., Cappello A.R., Fiermonte G., Dolce V., Capobianco L. (2023). Hyaluronic Acid: A Powerful Biomolecule with Wide-Ranging Applications-A Comprehensive Review. Int. J. Mol. Sci..

[10] Perez S., Makshakova O., Angulo J., Bedini E., Bisio A., de Paz J.L., Fadda E., Guerrini M., Hricovini M., Hricovini M. (2023). Glycosaminoglycans: What remains to be deciphered?. JACS Au.

[11] Ricard-Blum S., Vives R.R., Schaefer L., Gotte M., Merline R., Passi A., Heldin P., Magalhaes A., Reis C.A., Skandalis S.S. (2024). A biological guide to glycosaminoglycans: Current perspectives and pending questions. FEBS J..

[12] Ramanathan G., Hassan M., Rochev Y. (2024). Optimising the viscoelastic properties of hyaluronic acid hydrogels through colloidal particle interactions: A response surface methodology approach. Colloids Surf. A Physicochem. Eng. Asp..

[13] Saravanakumar K., Park S., Santosh S.S., Ganeshalingam A., Thiripuranathar G., Sathiyaseelan A., Vijayasarathy S., Swaminathan A., Priya V.V., Wang M.H. (2022). Application of hyaluronic acid in tissue engineering, regenerative medicine, and nanomedicine: A review. Int. J. Biol. Macromol..

[14] Hong G.W., Wan J., Park Y., Yoo J., Cartier H., Garson S., Haykal D., Yi K.H. (2024). Manufacturing Process of Hyaluronic Acid Dermal Fillers. Polymers.

[15] Misra S., Hascall V.C., Markwald R.R., Ghatak S. (2015). Interactions between Hyaluronan and Its Receptors (CD44, RHAMM) Regulate the Activities of Inflammation and Cancer. Front. Immunol..

[16] Tavianatou A.G., Caon I., Franchi M., Piperigkou Z., Galesso D., Karamanos N.K. (2019). Hyaluronan: Molecular size-dependent signaling and biological functions in inflammation and cancer. FEBS J..

[17] Bukhari S.N.A., Roswandi N.L., Waqas M., Habib H., Hussain F., Khan S., Sohail M., Ramli N.A., Thu H.E., Hussain Z. (2018). Hyaluronic acid, a promising skin rejuvenating biomedicine: A review of recent updates and pre-clinical and clinical investigations on cosmetic and nutricosmetic effects. Int. J. Biol. Macromol..

[18] Graca M.F.P., Miguel S.P., Cabral C.S.D., Correia I.J. (2020). Hyaluronic acid-Based wound dressings: A review. Carbohydr. Polym..

[19] Maloney F.P., Kuklewicz J., Corey R.A., Bi Y., Ho R., Mateusiak L., Pardon E., Steyaert J., Stansfeld P.J., Zimmer J. (2022). Structure, substrate recognition and initiation of hyaluronan synthase. Nature.

[20] Masselot--Joubert L., Di Renzo M.A. (2025). ATP-Binding Cassette (ABC) Transporters and Antibiotic Resistance: Specialized Systems for Capsular Polysaccharide Export in Gram-Negative Pathogens. Polysaccharides.

[21] Wang J., Wu Z., Cao L., Long F. (2024). Differential Regulation of Hyaluronan Synthesis by Three Isoforms of Hyaluronan Synthases in Mammalian Cells. Biomolecules.

[22] D’este M., Gennari G., Fidia Farmaceutici SpA (2021). Medicines for Topic Use Based on Sulfated Hyaluronic Acid as Activating or Inhibiting Agent of the Cytokine Activity. U.S. Patent.

[23] Zadnikova P., Sinova R., Pavlik V., Simek M., Safrankova B., Hermannova M., Nesporova K., Velebny V. (2022). The Degradation of Hyaluronan in the Skin. Biomolecules.

[24] Xu Y., Benedikt J., Ye L. (2024). Hyaluronic Acid Interacting Molecules Mediated Crosstalk between Cancer Cells and Microenvironment from Primary Tumour to Distant Metastasis. Cancers.

[25] Yang A., Yang P., Shen N., Wu R., Liu X., Ju Y., Lei L., Fang B. (2025). Bond-Centric Modifications of Hyaluronic Acid: Synthesis, Processing, and Biomedical Applications. Int. J. Nanomed..

[26] Buckley C., Murphy E.J., Montgomery T.R., Major I. (2022). Hyaluronic Acid: A Review of the Drug Delivery Capabilities of This Naturally Occurring Polysaccharide. Polymers.

[27] Galarraga J.H., Dhand A.P., Enzmann B.P., Burdick J.A. (2023). Synthesis, Characterization, and Digital Light Processing of a Hydrolytically Degradable Hyaluronic Acid Hydrogel. Biomacromolecules.

[28] Bokatyi A.N., Dubashynskaya N.V., Skorik Y.A. (2024). Chemical modification of hyaluronic acid as a strategy for the development of advanced drug delivery systems. Carbohydr. Polym..

[29] Grabowski M., Gmyrek D., Zurawska M., Trusek A. (2025). Hyaluronic Acid: Production Strategies, Gel-Forming Properties, and Advances in Drug Delivery Systems. Gels.

[30] Palencarova K., Koszagova R., Nahalka J. (2025). Hyaluronic Acid and Its Synthases-Current Knowledge. Int. J. Mol. Sci..

[31] Lim J., Machin D.R., Donato A.J. (2023). The role of hyaluronan in endothelial glycocalyx and potential preventative lifestyle strategy with advancing age. Current Topics in Membranes.

[32] Chylinska N., Maciejczyk M. (2025). Hyaluronic Acid and Skin: Its Role in Aging and Wound-Healing Processes. Gels.

[33] Monslow J., Govindaraju P., Puré E. (2015). Hyaluronan—A functional and structural sweet spot in the tissue microenvironment. Front. Immunol..

[34] Fedorova V.V., Tsitrina A., Halimani N., Kotelevtsev Y.V. (2025). 4-Methylumbelliferone, an Inhibitor of Hyaluronan Synthase, Prevents the Development of Oncological, Inflammatory, Degenerative, and Autoimmune Diseases. Biochemistry.

[35] La Gatta A., Stellavato A., Vassallo V., Di Meo C., Toro G., Iolascon G., Schiraldi C. (2021). Hyaluronan and Derivatives: An In Vitro Multilevel Assessment of Their Potential in Viscosupplementation. Polymers.

[36] Elvitigala K., Mubarok W., Sakai S. (2024). Hydrogels with Ultrasound-Treated Hyaluronic Acid Regulate CD44-Mediated Angiogenic Potential of Human Vascular Endothelial Cells In Vitro. Biomolecules.

[37] Khattak S., Ullah I., Yousaf M.T., Ullah S., Yousaf H., Li Y., Jin H., Shen J., Xu H.T. (2025). Advancements in hydrogels: A comprehensive review of natural, synthetic, and hybrid innovations for wound healing. Int. J. Biol. Macromol..

[38] Nimmo C.M., Owen S.C., Shoichet M.S. (2011). Diels-Alder Click cross-linked hyaluronic acid hydrogels for tissue engineering. Biomacromolecules.

[39] Verdoliva V., Bedini E., De Luca S. (2024). Sustainable Chemical Modification of Natural Polysaccharides: Mechanochemical, Solvent-Free Conjugation of Pectins and Hyaluronic Acid Promoted by Microwave Radiations. Biomacromolecules.

[40] He Q., Feng T., Xie Y., Swamiappan S., Zhou Y., Zhou Y., Zhou H., Peng X. (2025). Recent Advances in the Development and Application of Cell-Loaded Collagen Scaffolds. Int. J. Mol. Sci..

[41] Grieco M., Ursini O., Palama I.E., Gigli G., Moroni L., Cortese B. (2022). HYDRHA: Hydrogels of hyaluronic acid. New biomedical approaches in cancer, neurodegenerative diseases, and tissue engineering. Mater. Today Bio.

[42] Li S., Yao F., Liu Q., Tang C., Zhuo Y., Dai M., Lv Q., Zhong X. (2025). Natural polysaccharide hydrogels: Design, preparation, and tissue engineering applications. Mater. Des..

[43] Bu N., Li L., Hu X. (2023). Recent trends in natural polymer-based hydrogels for biomedical applications. Biofunct. Mater..

[44] Gholamali I., Vu T.T., Jo S.H., Park S.H., Lim K.T. (2024). Exploring the Progress of Hyaluronic Acid Hydrogels: Synthesis, Characteristics, and Wide-Ranging Applications. Materials.

[45] Del Olmo J.A., Alonso J.M., Martínez V.S., Ruiz-Rubio L., González R.P., Vilas-Vilela J.L., Pérez-Álvarez L. (2021). Biocompatible hyaluronic acid-divinyl sulfone injectable hydrogels for sustained drug release with enhanced antibacterial properties against Staphylococcus aureus. Mater. Sci. Eng. C.

[46] Hausen M.A., Moraes A.S., Pedrini F., Grabarz F., Camilli J.A., Duek E.A.R. (2024). Crosslinked Collagen-Hyaluronic Acid Scaffold Enhances Interleukin-10 Under Co-Culture of Macrophages And Adipose-Derived Stem Cells. Macromol. Biosci..

[47] Avila-Martinez N., Pfirrmann M., Gomes M., Krymchenko R., Versteeg E.M.M., Vlig M., Verdoes M., van Kuppevelt T.H., Boekema B., Daamen W.F. (2025). Effect of Hyaluronan in Collagen Biomaterials on Human Macrophages and Fibroblasts In Vitro. J. Funct. Biomater..

[48] Lee F., Chung J.E., Kurisawa M. (2008). An injectable enzymatically crosslinked hyaluronic acid- hydrogel system with independent tuning of mechanical strength and gelation rate. Soft Matter.

[49] Xu K., Narayanan K., Lee F., Bae K.H., Gao S., Kurisawa M. (2015). Enzyme-mediated hyaluronic acid-tyramine hydrogels for the propagation of human embryonic stem cells in 3D. Acta Biomater..

[50] Elvitigala K., Mohan L., Mubarok W., Sakai S. (2024). Phototuning of Hyaluronic-Acid-Based Hydrogel Properties to Control Network Formation in Human Vascular Endothelial Cells. Adv. Heal. Mater..

[51] Petit N., Chang Y.J., Lobianco F.A., Hodgkinson T., Browne S. (2025). Hyaluronic acid as a versatile building block for the development of biofunctional hydrogels: In vitro models and preclinical innovations. Mater. Today Bio.

[52] Lu J., Gao Z., He W., Lu Y. (2025). Harnessing the potential of hyaluronic acid methacrylate (HAMA) hydrogel for clinical applications in orthopaedic diseases. J. Orthop. Transl..

[53] Albtoush N., Petrey A.C. (2022). The role of hyaluronan synthesis and degradation in the critical respiratory illness COVID-19. Am. J. Physiol. Cell Physiol..

[54] Hu Y., Zhang Y., He J., Rao H., Wei Z., Shen Z., Zhou C. (2025). HMMR in human cancers: Regulatory mechanism and biological function. J. Transl. Med..

[55] Karalis T., Skandalis S.S. (2022). Hyaluronan network: A driving force in cancer progression. Am. J. Physiol. Cell Physiol..

[56] Hassn Mesrati M., Syafruddin S.E., Mohtar M.A., Syahir A. (2021). CD44: A Multifunctional Mediator of Cancer Progression. Biomolecules.

[57] Pedrycz-Wieczorska A., Chylinska-Wrzos P., Grzywacz A., Zielinski E., Bartosinski A., Kedziora-Kornatowska K., Lis-Sochocka M., Mertowska P., Mertowski S., Bojarski K. (2025). CD44 as a Central Integrator of Inflammation and Fibrosis: From Molecular Signaling to Environmental Modulation. Int. J. Mol. Sci..

[58] Wu S., Tan Y., Li F., Han Y., Zhang S., Lin X. (2024). CD44: A cancer stem cell marker and therapeutic target in leukemia treatment. Front. Immunol..

[59] Fujisawa S., Takagi K., Yamaguchi-Tanaka M., Sato A., Miki Y., Miyashita M., Tada H., Ishida T., Suzuki T. (2024). Receptor for Hyaluronan Mediated Motility (RHAMM)/Hyaluronan Axis in Breast Cancer Chemoresistance. Cancers.

[60] Demirel G., Cakil Y.D., Koltuk G., Aktas R.G., Caliskan M. (2024). The use of hyaluronic acid in a 3D biomimetic scaffold supports spheroid formation and the culture of cancer stem cells. Sci. Rep..

[61] Pibuel M.A., Poodts D., Molinari Y., Diaz M., Amoia S., Byrne A., Hajos S., Lompardia S., Franco P. (2023). The importance of RHAMM in the normal brain and gliomas: Physiological and pathological roles. Br. J. Cancer.

[62] Qi B., Musale V., Weng X., Banah A.K., Lawlor A., Murdoch C.E., Lay A.C., Heesom K.J., Coward R.J.M., O’Connor C.L. (2025). A Novel Role of Hyaluronan and Its Membrane Receptors, CD44 and RHAMM, in Obesity-Related Kidney Pathology. Biomolecules.

[63] Jackson D.G. (2019). Hyaluronan in the lymphatics: The key role of the hyaluronan receptor LYVE-1 in leucocyte trafficking. Matrix Biol..

[64] Lawrance W., Banerji S., Day A.J., Bhattacharjee S., Jackson D.G. (2016). Binding of Hyaluronan to the Native Lymphatic Vessel Endothelial Receptor LYVE-1 Is Critically Dependent on Receptor Clustering and Hyaluronan Organization. J. Biol. Chem..

[65] Ullah S., Zainol I. (2025). Fabrication and applications of biofunctional collagen biomaterials in tissue engineering. Int. J. Biol. Macromol..

[66] Xu F., Dawson C., Lamb M., Mueller E., Stefanek E., Akbari M., Hoare T. (2022). Hydrogels for Tissue Engineering: Addressing Key Design Needs Toward Clinical Translation. Front. Bioeng. Biotechnol..

[67] Wolf K.J., Kumar S. (2019). Hyaluronic Acid: Incorporating the Bio into the Material. ACS Biomater. Sci. Eng..

[68] Sahoo D.R., Biswal T. (2021). Alginate and its application to tissue engineering. SN Appl. Sci..

[69] Farshidfar N., Iravani S., Varma R.S. (2023). Alginate-Based Biomaterials in Tissue Engineering and Regenerative Medicine. Mar. Drugs.

[70] Islam M.M., Shahruzzaman M., Biswas S., Nurus Sakib M., Rashid T.U. (2020). Chitosan based bioactive materials in tissue engineering applications-A review. Bioact. Mater..

[71] Pramanik S., Aggarwal A., Kadi A., Alhomrani M., Alamri A.S., Alsanie W.F., Koul K., Deepak A., Bellucci S. (2024). Chitosan alchemy: Transforming tissue engineering and wound healing. RSC Adv..

[72] Ibrahim M.A., Alhalafi M.H., Emam E.M., Ibrahim H., Mosaad R.M. (2023). A Review of Chitosan and Chitosan Nanofiber: Preparation, Characterization, and Its Potential Applications. Polymers.

[73] Dovedytis M., Liu Z.J., Bartlett S. (2020). Hyaluronic acid and its biomedical applications: A review. Eng. Regen..

[74] Li L., He Z.Y., Wei X.W., Wei Y.Q. (2016). Recent advances of biomaterials in biotherapy. Regen. Biomater..

[75] Zhu Z., Wang Y.-M., Yang J., Luo X.-S. (2017). Hyaluronic acid: A versatile biomaterial in tissue engineering. Plast. Aesthetic Res..

[76] Zhu M., Lin S., Sun Y., Feng Q., Li G., Bian L. (2016). Hydrogels functionalized with N-cadherin mimetic peptide enhance osteogenesis of hMSCs by emulating the osteogenic niche. Biomaterials.

[77] Jeon S.O., Lamichhane S., Oh D.H., Seo J.E., Raj V., Lee S. (2025). Engineering a BMP2-risedronate complex with sustained release for osteoporosis therapy. Arch. Pharm. Res..

[78] Huang H., Feng J., Wismeijer D., Wu G., Hunziker E.B. (2017). Hyaluronic Acid Promotes the Osteogenesis of BMP-2 in an Absorbable Collagen Sponge. Polymers.

[79] Wang G., An Y., Zhang X., Ding P., Bi H., Zhao Z. (2021). Chondrocyte Spheroids Laden in GelMA/HAMA Hybrid Hydrogel for Tissue-Engineered Cartilage with Enhanced Proliferation, Better Phenotype Maintenance, and Natural Morphological Structure. Gels.

[80] Amann E., Wolff P., Breel E., van Griensven M., Balmayor E.R. (2017). Hyaluronic acid facilitates chondrogenesis and matrix deposition of human adipose derived mesenchymal stem cells and human chondrocytes co-cultures. Acta Biomater..

[81] Abedanzadeh M., Abolmaali S.S., Heidari R., Aalaei E., Kaviani M., Dara M., Mohammadi S., Azarpira N., Tamaddon A.M. (2024). Photo-crosslinked hyaluronic acid hydrogels designed for simultaneous delivery of mesenchymal stem cells and tannic acid: Advancing towards scarless wound healing. Int. J. Biol. Macromol..

[82] Dennaoui H., Chouery E., Rammal H., Abdel-Razzak Z., Harmouch C. (2018). Chitosan/hyaluronic acid multilayer films are biocompatible substrate for Wharton’s jelly derived stem cells. Stem Cell Investig..

[83] Kang L., Jia W., Li M., Wang Q., Wang C., Liu Y., Wang X., Jin L., Jiang J., Gu G. (2019). Hyaluronic acid oligosaccharide-modified collagen nanofibers as vascular tissue-engineered scaffold for promoting endothelial cell proliferation. Carbohydr. Polym..

[84] Xia Z., Guo B., Wu D., Yang F., Ding Y. (2024). Advances of natural hydrogel-based vascularization strategies for soft tissue repair. Front. Mater..

[85] Li J., Zhang K., Wu J., Liao Y., Yang P., Huang N. (2015). Co-culture of endothelial cells and patterned smooth muscle cells on titanium: Construction with high density of endothelial cells and low density of smooth muscle cells. Biochem. Biophys. Res. Commun..

[86] Lin Y., Huang C., Hou X., Kah J.C.Y., Wang J.W. (2025). Polydopamine and hyaluronic acid-coated dual-responsive silica nanoparticles for targeted atherosclerosis imaging and therapy. Nanoscale Adv..

[87] Murugesan M., Mathiyalagan R., Ramadhania Z.M., Nahar J., Luu C.H., Phan V.H.G., Yang D.C., Zhou Q., Chan Kang S., Thambi T. (2025). Tailoring hyaluronic acid hydrogels: Impact of cross-linker length and density on skin rejuvenation as injectable dermal fillers and their potential effects on the MAPK signaling pathway suppression. Bioact. Mater..

[88] Park H.J., Jin Y., Shin J., Yang K., Lee C., Yang H.S., Cho S.W. (2016). Catechol-Functionalized Hyaluronic Acid Hydrogels Enhance Angiogenesis and Osteogenesis of Human Adipose-Derived Stem Cells in Critical Tissue Defects. Biomacromolecules.

[89] Subramaniam S., Fang Y.H., Sivasubramanian S., Lin F.H., Lin C.P. (2016). Hydroxyapatite-calcium sulfate-hyaluronic acid composite encapsulated with collagenase as bone substitute for alveolar bone regeneration. Biomaterials.

[90] Senbanjo L.T., Chellaiah M.A. (2017). CD44: A Multifunctional Cell Surface Adhesion Receptor Is a Regulator of Progression and Metastasis of Cancer Cells. Front. Cell Dev. Biol..

[91] Wang K., Zhang T. (2016). Prognostic significance of CD168 overexpression in colorectal cancer. Oncol. Lett..

[92] Wang M., Deng Z., Guo Y., Xu P. (2022). Designing functional hyaluronic acid-based hydrogels for cartilage tissue engineering. Mater. Today Bio.

[93] Hashemi-Afzal F., Fallahi H., Bagheri F., Collins M.N., Eslaminejad M.B., Seitz H. (2025). Advancements in hydrogel design for articular cartilage regeneration: A comprehensive review. Bioact. Mater..

[94] Deng S., Cao H., Lu Y., Shi W., Chen M., Cui X., Liang J., Fan Y., Wang Q., Zhang X. (2024). Injectable dECM-enhanced hyaluronic microgels with spatiotemporal release of cartilage-specific molecules to improve osteoarthritic chondrocyte’s function. Collagen Leather.

[95] Krolikoski M., Monslow J., Pure E. (2019). The CD44-HA axis and inflammation in atherosclerosis: A temporal perspective. Matrix Biol..

[96] Parnigoni A., Viola M., Karousou E., Rovera S., Giaroni C., Passi A., Vigetti D. (2022). Hyaluronan in pathophysiology of vascular diseases: Specific roles in smooth muscle cells, endothelial cells, and macrophages. Am. J. Physiol. Cell Physiol..

[97] Tavakoli S., Evans A., Oommen O.P., Creemers L., Nandi J.B., Hilborn J., Varghese O.P. (2023). Unveiling extracellular matrix assembly: Insights and approaches through bioorthogonal chemistry. Mater. Today Bio.

[98] Pruzzo V., Bonomi F., Limido E., Weinzierl A., Harder Y., Laschke M.W. (2026). Injectable Scaffolds for Adipose Tissue Reconstruction. Gels.

[99] Chen K., Liu Y., Liu X., Guo Y., Liu J., Ding J., Zhang Z., Ni X., Chen Y. (2023). Hyaluronic acid-modified and verteporfin-loaded polylactic acid nanogels promote scarless wound healing by accelerating wound re-epithelialization and controlling scar formation. J. Nanobiotechnol..

[100] Rojano-Alfonso C., Lopez-Vicario C., Romero-Grimaldo B., Contreras B.J., Claria J., Titos E. (2025). Hyaluronic Acid in Liver Fibrosis: Role in Inflammation, Tissue Remodeling, and Disease Progression. Int. J. Mol. Sci..

[101] Marinho A., Nunes C., Reis S. (2021). Hyaluronic Acid: A Key Ingredient in the Therapy of Inflammation. Biomolecules.

[102] Highley C.B., Song K.H., Daly A.C., Burdick J.A. (2019). Jammed Microgel Inks for 3D Printing Applications. Adv. Sci..

[103] Chimene D., Lennox K.K., Kaunas R.R., Gaharwar A.K. (2016). Advanced Bioinks for 3D Printing: A Materials Science Perspective. Ann. Biomed. Eng..

